# Association of Household Utility of Cleaner Fuel With Lower Hypertension Prevalence and Blood Pressure in Chinese Adults

**DOI:** 10.3389/ijph.2022.1605193

**Published:** 2022-11-24

**Authors:** Zhiguang Liu, Mengya Li, Yibing Zhu, Perry Hystad, Yuanting Ma, Sumathy Rangarajan, Qian Zhao, Lihua Hu, Salim Yusuf, Yang Li, Lap Ah Tse

**Affiliations:** ^1^ Department of Pharmacy and Clinical Trial Unit, Beijing Anzhen Hospital, Capital Medical University, Beijing, China; ^2^ Medical Research and Biometrics Center, National Clinical Research Center for Cardiovascular Diseases, Fuwai Hospital, National Center for Cardiovascular Diseases, Peking Union Medical College and Chinese Academy of Medical Sciences, Beijing, China; ^3^ Department of Emergency, Guang’anmen Hospital, China Academy of Chinese Medical Sciences, Beijing, China; ^4^ School of Biological and Population Health Sciences, College of Public Health and Human Sciences, Oregon State University, Corvallis, OR, United States; ^5^ Dongguan street Community Health Service Center, Xining, China; ^6^ Population Health Research Institute, McMaster University and Hamilton Health Sciences, Hamilton, ON, Canada; ^7^ General Practice Ward/International Medical Center Ward, General Practice Medical Center, West China Hospital, Sichuan University, Chengdu, Sichuan, China; ^8^ Nanchang Center for Disease Control and Prevention, Nanchang, China; ^9^ JC School of Public Health and Primary Care, The Chinese University of Hong Kong, Hong Kong, Hong Kong SAR, China

**Keywords:** hypertension, air pollution, biomass, household fuel use, solid fuel, energy ladder, cleanliness

## Abstract

**Objectives:** To investigate whether lower hypertension prevalence or blood pressure was associated with cleaner household fuel usage for cooking and heating among Chinese adults.

**Methods:** We enrolled 44,862 Chinese adults at the baseline of the prospective urban and rural epidemiology (PURE) study in China during 2005–2009, as a subset of the PURE-global China site. Multilevel logistic regression and generalized linear mixed models were conducted to estimate the adjusted odds ratio (AOR) and regression coefficient for hypertension and blood pressure respectively, while subgroup analysis by ambient PM2.5 concentration and location was also examined.

**Results:** Compared with the least clean household solid fuel group, gas (AOR = 0.91, 95% CI: 0.83, 0.99) or electricity (AOR = 0.72, 95% CI: 0.60, 0.87) was associated with significantly lower levels of hypertension prevalence and blood pressure, and a similar pattern of the association was consistently observed among participants with high ambient PM2.5 exposure and those living in urban areas.

**Conclusion:** Household utility of cleaner fuel type was associated with lower hypertension prevalence and blood pressure in Chinese adults. Our study urges the utilization of cleaner household energy to mitigate the burden of hypertension.

## Introduction

Growing epidemiological evidence has shown that air pollution is one of the most important risk factors for increasing the global burden of cardiovascular diseases and all-cause mortality [1–5]. Current research demonstrated that household air pollution generated from burning solid fuel could cause more deaths than that of the ambient air pollution (3.5 million vs. 3.1 million) [6]. Despite household facilities for cooking and heating having been greatly improved in recent years [7, 8], household air pollution remains the leading risk factor associated with increased mortality in the east of Asia [5, 9]. Previous epidemiological studies in South America, Africa, India, and China have investigated the association between household air pollution emitted from burning household solid fuels for cooking and hypertension prevalence, using the clean fuels as a comparison (i.e., combined gas and electricity), but obtained inconsistent findings [10–14]. Few of them specified the individual fuel type for cooking according to energy efficiency, classified by the level of cleanliness of the fuel determined by their potential emission amount of particulate matters during combustion, i.e., from solid fuel (the lowest cleanliness), liquid, gas, to electricity (the highest cleanliness) [15], while the association with household fuel uses for heating were not specifically addressed in the previous studies. Household gas fuel is widely regarded as clean energy, however, results from an experimental study revealed that the median concentration of PM2.5 was substantially higher in kitchens using gas-fueled stoves than in kitchens using electric induction cookers (0.281 mg/m^3^ vs. 0.155 mg/m^3^) [16]. To date, evidence has been lacking on the association of hypertension or blood pressure with different types of household fuel use for cooking and heating. Moreover, it is likely that the hazardous effect of utilizing less clean household fuels on hypertension and blood pressure may become stronger among residents with relatively high ambient PM2.5 exposure, resulting from cumulative inflammation and injury in the vascular endothelium induced by the toxic effects of the indoor and outdoor PM2.5 air pollution [17]. Nevertheless, this potential interaction between ambient PM2.5 and different household fuel uses has been attempted to a lesser extent in the previous epidemiological studies. To address these knowledge gaps, this study aimed to investigate whether higher hypertension prevalence or blood pressure was associated with less clean household fuel usage for cooking and heating in a large sample of Chinese adults. In addition, whether these associations were modified by ambient PM2.5 exposure was also examined.

## Methods

### Study Subject and Design

Participants of this report are part of the Prospective Urban and Rural Epidemiology (PURE) study, who were recruited as the baseline in China during 2005–2009 via a three-level cluster sampling approach (center, community, and household) [18]. PURE-China included a total of 47,677 subjects from 115 communities in 12 centers (i.e., Yunnan, Qinghai, Beijing etc.), and the selection of communities was based on the feasibility for long-term follow-up of study participants with less residential mobility (i.e., living in their current home for a further 4 years) [19]. A total of 2,815 participants were excluded due to missing information on household fuel use for cooking or heating, diagnostic status of hypertension, and age at the time of recruitment. Finally, 44,862 eligible participants were retained in the analysis. The PURE-China study was approved by an institutional ethics review committee and the written informed consent was obtained from each participant before the fieldwork was initiated.

### Procedures and Data Collection

Trained research staff conducted face to face interviews using standard questionnaires and a physical examination was performed on each eligible participant. Briefly, we invited each participant to complete a standard questionnaire including information on socio-demographic level (age, sex, location, center, occupation, education attainment, and household possession), lifestyle behavior factors (smoking and alcohol status, secondhand smoke exposure, physical activity, and dietary energy intake), medical history, anti-hypertensive drug, and household fuel use for primary cooking or heating fuel. The physical examination included anthropometric measures (weight, height) and resting blood pressure measurement (sitting) according to standard protocols. The location of each participant’s residence was defined as rural or urban according to geographical location. The community is defined as a group of people who have common characteristics and reside in a defined geographical area. Rural communities represent villages more than 50 km away from urban communities or without easy access to commuter transportation [20, 21]. Centers refer to a province of China, so this study has 12 centers. Education attainment was categorized as high (trade school, college or university), middle (secondary or high school), or low (primary or no education). An ever cigarette smoker or alcohol drinker was defined as an individual who was currently using or had ever used any tobacco or alcohol products at the time of interview, otherwise, they were classified as a non-smoker or non-alcohol drinker [22, 23]. Secondhand smoke exposure was defined if an individual had ever lived or worked with at least one smoker and it was categorized as yes or no. Physical activity was assessed using the International Physical Activity Questionnaire and was categorized as low (<600 MET-minutes per week), moderate (600–3000 MET-minutes per week), and high (≥3000 MET-minutes per week). Body mass index (BMI) was calculated by body weight (kilogram) divided by the square of the body height and then it was further categorized as underweight (BMI<18.5), normal (18.5≤BMI<24), overweight (24≤BMI<28), and obese (BMI≥28) [24]. Daily energy intake was assessed using a validated Food Frequency Questionnaire [25]. Household wealth index was calculated according to household possession consisting of 14 assets, which could partly reflect household wealth status. Principal components analysis was then performed to identify the factor with the largest Eigen value, and the factor loading (ranging from −2.25 to 1.75) was treated as weight for each manifest variable when calculating the household wealth index [26]. Each participant’s medical history referred to those who had ever been diagnosed as any of the following diseases: diabetes, stroke, heart failure, other heart diseases, or cancer. History of anti-hypertensive drug treatment was acquired from all participants and it was categorized as “received” and “never received.”

Annual PM2.5 concentrations were estimated based on satellite and fixed monitoring data by using a geographically weighted regression model with a resolution of 1 km*1 km. To estimate aerosol optical depth, multiple satellite products were analyzed and combined with data from the Sun Photometer and GEOS-Chem simulations [8]. These estimates were used to predict ground-level annual PM2.5 concentrations for each community of PURE study. All people living in the same community were assigned the same concentration of PM2.5 [27].

### Outcome Assessment of Hypertension

We adopted a standard and calibrated digital blood pressure measuring device (Omron HEM-757; Omron Healthcare Co. Ltd., Scarborough, Ontario, Canada) with a cuff size of 14 × 48 cm supplied to measure sitting blood pressure twice separately on the participant’s right arm 1 min apart [28, 29]. We took the average value of two separate measurements of systolic blood pressure (SBP) and diastolic blood pressure (DBP) for further analysis. Participants were asked to take at least 5 min to rest and wait 30 min after smoking, exercising, eating, or climbing stairs before measuring blood pressure [18, 30]. A participant was defined as having hypertension if their mean value of SBP≥140 mmHg or DBP≥90 mmHg was obtained at the baseline survey, they were receiving anti-hypertensive drug treatment, or they self-reported hypertension or a physician diagnosed hypertension.

### Exposure Assessment of Household Fuel Uses

Information of primary household fuel use was collected at the household level using a standard questionnaire, and participants from the same household were assumed to use the same fuel for cooking and heating. Detailed methods of data collection of primary household fuel use were reported previously [20]. Briefly, we collected information of primary types of household fuel use for cooking and heating via face-to-face interviews. We classified them as 4 major types of utility according to their ranking in energy efficiency classified from the lowest to the highest class of cleanliness determined by their potential emission amount of particulate matters from the combustion process [31]. Electricity does not involve combustion at the household level, it was regarded as the stringent clean fuel and thus was ranked in the highest class of cleanliness on the energy ladder. Combustion of solid fuel emits the highest amounts of particulate matters and thus was ranked as the fuel with the lowest efficiency and the lowest class of cleanliness on the energy ladder, followed by the middle and relatively higher efficient energy sources, i.e., liquid fuel and gas fuel.

We adopted the following steps to regroup types of household fuel utility for cooking or heating based on the cleanliness level of fuel. Step 1, we classified specific types of fuel use for cooking and heating as electricity only (stringent clean energy), gas (gas or gobar gas), liquid (kerosene), and solid (harcoal, coal, wood, agriculture straw, animal dung, and shrub). Step 2, we further categorized portable heaters into liquid as these mainly use kerosene, and open fires into solid as their main sources were coal, wood, or agriculture as heating fuel. We combined central heating with electricity, as central heating is facilitated via transporting heating from other places through a central pipeline without involving any combustion and thus it is also regarded as the stringent clean fuel as well. We also combined participants without fuel use for heating into the electricity category for heating, as they did not use any method that involved combustion and no emission of PM2.5 was generated. Step 3, we defined a clean group for household fuel use if electricity was adopted for both the cooking and heating processes. The gas fuel group for household fuel use referred to participants used gas for cooking or heating but did not involve a lower class energy source (i.e., liquid or solid fuel). The classification of the liquid fuel group only counted the use of liquid fuel for either cooking or heating, but did not involve the lowest class energy source (i.e., solid fuel). Solid fuel group referred to participants who adopted solid fuel for either cooking or heating fuel regardless of whether a higher class fuel type in energy ladder was also used.

### Statistical Analysis

We reported categorical variables as frequency and proportion, while continuous variables were described as mean and standard deviation (SD) for normal distribution and median (and interval of quantile range, IQR) for skewed distribution. The association between primary household fuel usage by energy cleanliness and hypertension prevalence were examined using a multilevel logistic regression model after taking the center as a random effect to deal with the potential clustering effect of participants within the centers. Base model was adjusted for age, sex, and location, the full model was further controlled for smoking status, secondhand smoke exposure, drinking status, education level, occupational class, household wealth index, BMI status, physical activity, energy intake, and ambient PM2.5 concentration. We investigated the associations between types of primary household fuel use and blood pressure by using a generalized linear mixed model with random intercepts to account for clustering by centers. The base model for blood pressure was the same as that of hypertension, and the full model was also same as that of hypertension but further adjusted for anti-hypertensive drug treatment and medical history.

We explored the association between primary types of household fuel uses and hypertension prevalence or blood pressure according to ambient PM2.5 exposure (high and low, categorized by median value) and location of residence (urban or rural) to understand the potential interaction. Furthermore, an interaction term (household fuel use × anti-hypertensive drug treatment) was introduced into the full model and sensitivity analyses were also performed among participants with and without receiving any anti-hypertensive drug treatment to see if the association between household fuel use and blood pressure was robust. All of the statistical analysis was conducted by SAS® software (version 9.4) with a significance level of 0.05 using two-sided test.

## Results

A total of 44,862 eligible participants were included in this report, with a mean age of 51.1 years old (SD = 9.7) and women making up 58.4%. The annual median concentration of ambient PM2.5 was 45.8 ug/m^3^, and 50.8% of the participants inhabited a rural area. 25,336 (56.5%) of the participants were classified into the solid fuel use group, while 974 (2.2%) and 17,534 (39.1%) were classified as the liquid fuel use and gas fuel use group for either cooking or heating, respectively. There were 685 participants using electricity as the primary household fuel for both heating and cooking.

Table 1 summarizes the distribution of basic characteristics of 44,862 participants using different household fuel types according to the cleanliness of fuel. Among all primary types of fuel users, participants who adopted solid fuel as their primary source for heating or cooking were more likely to inhabit a rural area, have a lower household wealth index value, consume higher amounts of energy, be in the normal weight category, and have relatively higher SBP or DBP. Participants in the liquid fuel use subgroup had relatively lower SBP and DBP. More participants in the gas fuel use subgroup received high education, had a higher wealth index value, and were more prone to be exposed to lower ambient PM2.5, however, participants in the electricity subgroup were more prone to exposed to higher ambient PM2.5, alcohol consumption, and were more likely to be overweight or obese.

**TABLE 1:** Basic characteristics of the 44,862 study participants by primary household fuel use (Prospective Urban and Rural Epidemiology study in China, 2005–2009).

	All participants	Solid	Liquid	Gas	Electricity
No. (%)	44,862 (100)	25,336 (56.5)	974 (2.2)	17,534 (39.1)	1,018 (2.3)
Age, mean ± SD, y	51.1 ± 9.65	50.1 ± 9.48	53.3 ± 9.65	52.4 ± 9.68	51.5 ± 9.87
Female (n, %)	26,200 (58.4)	14,435 (57)	588 (60.4)	10,573 (60.3)	604 (59.3)
Socioeconomic status					
Rural (n, %)	22,776 (50.8)	20,212 (79.8)	189 (19.4)	2,199 (12.5)	176 (17.3)
Education attainment					
Primary (n, %)	15,204 (34)	11,312 (44.8)	206 (21.2)	3,262 (18.7)	424 (41.7)
Secondary (n, %)	22,916 (51.2)	11,962 (47.3)	552 (56.7)	9,955 (56.9)	447 (44)
College/University (n, %)	6,620 (14.8)	1993 (7.9)	215 (22.1)	4,267 (24.4)	145 (14.3)
Household wealth index (n, %)					
Lower	14,799 (33)	12,714 (50.2)	114 (11.7)	1722 (9.8)	249 (24.5)
Middle	15,190 (33.9)	8,001 (31.6)	363 (37.3)	6,452 (36.8)	374 (36.7)
Higher	14,872 (33.2)	4,620 (18.2)	497 (51)	9,360 (53.4)	395 (38.8)
Unhealthy lifestyle habits					
Ever smoking (n, %)	12,079 (27.4)	7,092 (28.5)	219 (22.9)	4,488 (25.9)	280 (28.2)
Secondhand smoke exposure (n, %)	13,055 (38.8)	7,261 (39.1)	254 (32.7)	5,281 (39)	259 (33.9)
Ever alcohol drinking (n, %)	10,835 (24.4)	6,349 (25.4)	210 (21.7)	4,003 (23)	273 (27.4)
Physical activity (MET score)					
Low (<600) (n, %)	7,467 (16.6)	4,692 (18.5)	239 (24.5)	2,306 (13.2)	230 (22.6)
Moderate (600 to <3,000) (n, %)	18,705 (41.7)	10,541 (41.6)	459 (47.1)	7,302 (41.6)	403 (39.6)
High (≥3,000) (n, %)	18,690 (41.7)	10,103 (39.9)	276 (28.3)	7,926 (45.2)	385 (37.8)
Energy intake (n, %)					
Lower	15,915 (35.5)	8,381 (33.1)	430 (44.1)	6,835 (39)	269 (26.4)
Middle	14,389 (32.1)	8,112 (32)	287 (29.5)	5,630 (32.1)	360 (35.4)
Higher	14,558 (32.5)	8,843 (34.9)	257 (26.4)	5,069 (28.9)	389 (38.2)
Physical measurement					
BMI, mean ± SD, kg/m^2^	24.6 ± 3.66	24.5 ± 3.74	24.6 ± 3.56	24.7 ± 3.56	24.8 ± 3.45
Underweight (n, %)	1,488 (3.3)	932 (3.7)	33 (3.4)	495 (2.8)	28 (2.8)
Normal weight (n, %)	19,518 (43.5)	11,386 (44.9)	414 (42.5)	7,306 (41.7)	412 (40.5)
Overweight (n, %)	16,741 (37.3)	8,974 (35.4)	373 (38.3)	6,984 (39.8)	410 (40.3)
Obese (n, %)	7,115 (15.9)	4,044 (16)	154 (15.8)	2,749 (15.7)	168 (16.5)
SBP, mean ± SD, mmHg	133.6 ± 22.42	135.6 ± 22.61	128.7 ± 21.48	131.4 ± 21.94	129.2 ± 21.71
DBP, mean ± SD, mmHg	82.9 ± 13.21	83.8 ± 13.7	80.6 ± 12.08	81.7 ± 12.45	80.7 ± 12.61
Other exposures					
Medical history	3,813 (8.5)	1711 (6.8)	97 (10)	1915 (10.9)	90 (8.8)
With anti-hypertension medicine (n, %)	6,023 (13.4)	2,766 (10.9)	169 (17.4)	2,968 (16.9)	120 (11.8)
Ambient PM_2.5,_ median, IQR, ug/m^3^	45.8 (39.9)	45.8 (51.3)	49.2 (25.4)	43.8 (24.2)	53.9 (34.2)

BMI, body mass index; SD, standard deviation; IQR, interquartile range; SBP, systolic blood pressure; DBP, diastolic blood pressure.

Underweight: BMI<18.5, Normal: 18.5≤BMI<24, Overweight: 24≤BMI<28, Obese: BMI≥28.

Table 2 compares the associations of hypertension prevalence and blood pressure with different household fuel uses for cooking or heating based on the cleanliness level of fuel. Using the least clean “solid fuel” as the reference, participants who adopted cleaner fuels (i.e., gas and electricity) were associated with significantly lower hypertension prevalence after a full adjustment of a variety of confounding factors, except for the household liquid fuel users. Utilizing higher class energy efficient fuel types tended to be associated with a lower risk of SBP or DBP, with the most prominent effect for participants using electricity as the primary household fuel.

**TABLE 2:** Associations of household fuels according to cleanliness ranking on the energy ladder with hypertension prevalence and blood pressure (Prospective Urban and Rural Epidemiology study in China, 2005–2009).

Fuel uses	No. of hypertension	Prevalence crude rate (95%CI)	Hypertension (AOR, 95% CI)		Systolic blood pressure (mmHg, 95% CI)		Diastolic blood pressure (mmHg, 95% CI)	
			Model 1^a^	Model 2^b^	Model 1^a^	Model 2^c^	Model 1^a^	Model 2^c^
Solid	10,902 (58.1)	43.0 (42.42, 43.64)	1.00	1.00	Ref	Ref		
Liquid	376 (2.00)	38.6 (35.55, 41.66)	0.97 (0.84, 1.12)	0.95 (0.78, 1.15)	**−2.68 (-4.07, −1.28)**	**−2.83 (−4.46, -1.20)**	**−**0.24 (**−**1.12, 0.63)	**−**0.87 (**−**1.82, 0.08)
Gas	7,114 (37.9)	40.6 (39.85, 41.30)	0.97 (0.91, 1.03)	**0.91 (0.83, 0.99)**	**-1.57 (-2.15, -0.98)**	**−2.40 (−3.10, −1.71)**	**−**0.03 (**−**0.39, 0.34)	**−0.52 (−0.94, −0.09)**
Electricity	372 (2.00)	36.5 (33.58, 39.50)	**0.80 (0.69, 0.92)**	**0.72 (0.60, 0.87)**	**−3.42 (−4.79, −2.04)**	**−5.26 (−6.84, −3.67)**	**−1.99 (−2.85, -1.13)**	**−2.35 (−3.32, -1.38)**
*p* value (for trend)			**0.04**	**<0.01**	**<0.01**	**<0.01**	0.09	**<0.01**

^a^ Model 1 adjusted for age, sex, location, and random effect for center.

^b^ Model 2 adjusted for age, sex, location, smoking status, secondhand smoke exposure, drinking status, education level, occupational class, household wealth index, BMI status, physical activity, energy intake, ambient PM2.5 concentration, and random effect for center.

^c^ Model 2 adjusted for age, sex, location, smoking status, secondhand smoke exposure, drinking status, education level, occupational class, household wealth index, BMI status, physical activity, energy intake, personal medical history, ambient PM2.5 concentration, taking hypertension medicine, and random effect for center.

Table 3 presents the association between hypertension prevalence, blood pressure, and primary fuel use in both the high and low ambient PM2.5 exposure groups. A significant interaction was observed between household fuel use and ambient PM2.5 exposure on the effect of SBP (*p* < 0.001) or DBP (*p* = 0.02). Among the subgroup with high ambient PM2.5 exposure, compared with the solid fuel subgroup, participants using a cleaner fuel tended to be associated with lower risk of hypertension and blood pressure, specifically for the electricity group (AOR = 0.71, 95% CI: 0.58, 0.87; SBP = −6.08, 95%CI: −7.86, −4.31; DBP = −2.62, 95% CI: −3.75, −1.49). In the low ambient PM2.5 exposure group, a significantly lower odds with blood pressure was only observed among those utilizing gas as the primary fuel. The pattern of association among participants inhabiting urban and rural areas was similar to those with high and low ambient PM2.5 exposure, respectively (Table 4). The association was more significant in the urban subgroup.

**TABLE 3:** The association between hypertension prevalence/blood pressure and energy ladder, stratified by ambient Particulate Matter 2.5^a^ (Prospective Urban and Rural Epidemiology study in China, 2005–2009).

	Low ambient PM2.5 (N = 24,115)				High ambient PM2.5 (N = 20,747)			
Fuel type	No. of hypertension	AOR^b^ (95% CI)	SBP^c^ (mmHg, 95% CI)	DBP^c^ (mmHg, 95% CI)	No. of hypertension	AOR^b^ (95% CI)	SBP^c^ (mmHg, 95% CI)	DBP^c^ (mmHg, 95% CI)
Solid	5,354 (40.5)	1.00	Ref		5,548 (45.8)	1.00	Ref	
Liquid	205 (36.4)	0.95 (0.75, 1.22)	−1.18 (−3.15, 0.78)	−0.37 (−1.62, 0.88)	171 (41.6)	0.84 (0.65, 1.09)	**−4.97 (−7.31, −2.64)**	**−1.83 (−3.32, −0.34)**
Gas	4,104 (40.4)	0.99 (0.88, 1.10)	**−1.10 (−2.01, -0.19)**	−0.51 (−1.10, 0.07)	3,010 (40.8)	**0.85 (0.76, 0.95)**	**−2.86 (−3.85, -1.87)**	**−0.62 (−1.25, −0.01)**
Electricity	56 (31.8)	0.95 (0.61, 1.46)	1.45 (−2.02, 4.92)	0.22 (−1.99, 2.43)	316 (37.5)	**0.71 (0.58, 0.87)**	**−6.08 (−7.86, −4.31)**	**−2.62 (−3.75, −1.49)**
*p* value (for trend)		0.99	**0.05**	0.12		**<0.01**	**<0.01**	**<0.01**

^a^ Low and high ambient PM2.5 was classified by median value (50.8 ug/m^3^).

^b^ Model adjusted for age, sex, location, smoking status, secondhand smoke exposure, drinking status, education level, occupational class, household wealth index, BMI status, physical activity, energy intake, and random effect for center.

^c^ Model adjusted for age, sex, location, smoking status, secondhand smoke exposure, drinking status, education level, occupational class, household wealth index, BMI status, physical activity, energy intake, personal medical history, taking hypertension medicine, and random effect for center.

SBP, systolic blood pressure; DBP, diastolic blood pressure.

**TABLE 4:** The association between hypertension prevalence/blood pressure and energy ladder, stratified by location (Prospective Urban and Rural Epidemiology study in China, 2005–2009).

Fuel type	Rural areas (N = 22,776)				Urban areas (N = 22,086)			
	No. of hypertension	AOR^a^ (95% CI)	SBP^b^ (mmHg, 95% CI)	DBP^b^ (mmHg, 95% CI)	No. of hypertension	AOR^a^ (95% CI)	SBP^b^ (mmHg, 95% CI)	DBP^b^ (mmHg, 95% CI)
Solid	8,574 (42.4)	1.00	Ref		2,328 (45.4)	1.00	Ref	
Liquid	71 (38.1)	0.78 (0.52, 1.16)	−2.29 (−5.64, 1.07)	−0.66 (−2.86, 1.54)	304 (38.7)	0.94 (0.75, 1.18)	−**2.69 (**−**4.62,** −**0.77)**	−0.67 (−1.86, 0.53)
Gas	803 (36.5)	0.87 (0.75, 1.00)	−0.45 (−1.74, 0.84)	−0.22 (−1.06, 0.63)	6,311 (41.2)	0.94 (0.84, 1.05)	−**2.45 (**−**3.38, −1.52)**	**−0.91 (-1.49, -0.34)**
Electricity	77 (43.8)	0.97 (0.64, 1.47)	−3.25 (−6.85, 0.36)	0.57 (−1.79, 2.94)	295 (35.0)	**0.78 (0.63, 0.98)**	−**2.86 (**−**4.70,** −**1.02)**	−**2.23 (**−**3.38,** −**1.09)**
*p* value (for trend)		0.06	0.13	0.78		0.08	**<0.01**	**<0.01**

^a^ Model adjusted for age, sex, smoking status, secondhand smoke exposure, drinking status, education level, occupational class, household wealth index, BMI status, physical activity, energy intake, ambient PM2.5 concentration, and random effect for center.

^b^ Model adjusted for age, sex, smoking status, secondhand smoke exposure, drinking status, education level, occupational class, household wealth index, BMI status, physical activity, energy intake, personal medical history, ambient PM2.5 concentration, taking hypertension medicine, and random effect for center.

SBP, systolic blood pressure; DBP, Diastolic blood pressure.A.

Sensitivity analyses revealed that participants not receiving anti-hypertensive drug treatment showed a significantly lower trend for the association of blood pressure with the rising cleanliness ranking of fuel types, and a significant interaction between household fuel use and anti-hypertensive medication was suggested for the risk of blood pressure (*p* < 0.001) (Table 5).

**TABLE 5:** The association between blood pressure and energy ladder, stratified by anti-hypertension medicine^a^ (Prospective Urban and Rural Epidemiology study in China, 2005–2009).

Fuel type	Without anti-hypertension medicine (N = 38,839)			With anti-hypertension medicine (N = 6,023)		
	No. of hypertension	SBP (mmHg, 95% CI)	DBP (mmHg, 95% CI)	No. of hypertension	SBP (mmHg, 95% CI)	DBP (mmHg, 95% CI)
Solid	8,184 (36.3)	Ref		2,718 (98.3)	Ref	
Liquid	209 (26.0)	**−3.09 (−4.68, −1.50)**	−0.56 (−1.58, 0.47)	167 (98.8)	**−6.94 (−11.1, −2.82)**	**−3.16 (−5.60, −0.71)**
Gas	4,186 (28.7)	**−2.46 (−3.15, −1.76)**	**−0.46 (−0.91, −0.01)**	2,928 (98.7)	−1.40 (−3.40, 0.60)	−1.08 (−2.26, 0.11)
Electricity	253 (28.2)	**−5.45 (−7.05, −3.86)**	**−2.55 (−3.58, −1.52)**	119 (99.2)	−1.35 (−5.96, 3.27)	−1.86 (−4.58, 0.89)
*p* value (for trend)		**<0.01**	**<0.01**		0.40	0.10

^a^ Model adjusted for age, sex, location, smoking status, secondhand smoke exposure, drinking status, education level, occupational class, household wealth index, BMI status, physical activity, energy intake, personal medical history, ambient PM2.5 concentration, and random effect for center.

SBP, systolic blood pressure; DBP, diastolic blood pressure.

## Discussion

This was a population-based multi-center study demonstrating that utilizing cleaner household fuel was associated with lower prevalence of hypertension and a lower level of blood pressure. Electricity is the stringent clean fuel source as it does not involve a combustion process or emission of particulate matters. Compared with the least clean solid fuel, participants using electricity fuel for household cooking and heating had relatively lower risks of hypertension prevalence and a lower level of blood pressure than those using the liquid or gas fuel, and the findings were more prominent among participants not receiving anti-hypertensive medications, despite a consistent pattern that was also observed among those receiving anti-hypertensive drug treatment. A consistently decreasing trend for the association between hypertension or blood pressure and the rising cleanliness of household fuel was demonstrated among participants exposed to a relatively high ambient PM2.5 concentration and those inhabiting urban areas where the outdoor air population was also high, and these novel findings have never been reported in previous studies.

Except for this PURE-China study, only a few epidemiological studies investigated the association between household fuel use and hypertension prevalence in China [22, 23, 32–34]. A hospital-based study containing 14,068 adults in Shanghai of China showed a significantly increased risk of hypertension (OR = 1.70, 95% CI = 1.40, 2.07) among ever solid fuel users [32]. Another study including 9 regions of China with 4,594 participants also reported a similar finding that indoor air pollution emitted from using household solid fuel for cooking or heating was significantly associated with hypertension prevalence (OR = 1.11, 95% CI = 1.11, 1.12) [33]. Deng et al reported that biomass fuel for cooking was associated with a slightly higher risk of hypertension based on 3,754 older Chinese adults [23]. In addition, a study of 8,067 elderly participants over 65 years in China showed that people using solid fuel for cooking may be at a higher risk of elevated blood pressure than those using clean fuel, but no association between solid fuel use and hypertension was observed [22]. However, these previous studies only took cooking fuel into account, without considering heating fuel. A recent study demonstrated that indoor solid fuel use for heating was associated with increases in SBP or DBP levels and increased risk of hypertension, but no significant association was observed between indoor fuel use for cooking and hypertension or blood pressure [8]. Overall, a literature review showed that none of the previous studies provided evidence on the association with other major types of household fuel use according to the level of cleanliness, and the residual confounding effect is still a concern. Compared with the previous studies, our study addressed more potential confounding factors, such as education level, occupational class, secondhand smoke exposure, energy intake, and ambient PM2.5 concentration. A global study analyzed 12 Demographic and Health Surveys (DHS) from 10 countries (i.e., Albania, Armenia, Azerbaijan, Bangladesh, Benin, Ghana, Kyrgyzstan, Lesotho, Namibia, and Peru), by using clean cooking fuel (including gas) as the reference group, they found that using household solid fuel was not significantly associated with hypertension prevalence (OR = 1.07, 95%CI = 0.99, 1.16) [10]. Our study used the solid fuel as the comparison and revealed a significantly lower odds between using electricity as household fuel and hypertension prevalence (AOR = 0.72, 95% = 0.60, 0.87), as well as a significantly lower level of SBP by5.26 mmHg and DBP by 1.99 mmHg. Gas was regarded as a clean fuel in many previous studies when comparing the health outcomes with household solid fuel use [20, 35]; however, field measurement results indicated that gas during the combustion process could still increase the concentrations of several hazardous chemical pollutants including Carbonic oxide, PM2.5, nitric oxide, and nitrogen dioxide [36]. Our analysis also found significantly lower odds of hypertension of 0.91 and a significantly lower level of SBP or DBP by 2.40 mmHg or 0.52 mmHg for participants using gas as their primary household fuel for cooking and heating; nevertheless, these risks were still about 5%–28% higher than for those using electricity as the primary household fuel. Therefore, evidence from our study highlights that using household gas fuel as the benchmark of clean fuel may lead to an underestimation of the actual effect of other types of household fuel on the risk of hypertension or blood pressure in the previous studies [37].

Few of the previous epidemiological studies compared the effect of specific fuel type classified based on cleanliness and fuel efficiency on the risk of hypertension and blood pressure levels between groups with high and low ambient PM2.5 exposure. Our study demonstrated significantly lower odds between using cleaner household fuels and hypertension or a lower blood pressure level in participants with high ambient PM2.5 exposure, with the lowest association among those utilizing electricity as the primary household fuel for cooking and heating. A similar pattern was also suggested among people inhabiting urban areas in which the air pollution level was higher than the rural areas. Long-term exposure to ambient PM2.5 may increase blood C-reactive protein and oxidative stress, causing systemic inflammation, which in turn may lead to atherosclerosis and adversely alter vascular functions [38]. Such chronic systemic inflammation in the circulation system may be exaggerated by further exposure to indoor PM2.5 emitted from combustion of household fuels for cooking and heating, and this biological mechanism reasonably explains the observed lower odds of hypertension or lower blood pressure levels with increasing cleanliness of the household fuel.

### Strengths and Limitations

The strengths of this study include it being a global standardized study design with good quality control, large sample size, good representation of participants with diverse social economic backgrounds, and standardized approaches to data collection via a detailed questionnaire. Importantly, this is the first Chinese study characterizing the health effects of household air pollution derived from fuel usage based on order of cleanliness, and thus is the first to present the scientific evidence that moving up the energy ladder is associated with lower hypertension prevalence and blood pressure level in Chinese adults. Moreover, we applied random effect into the model which fully considered the heterogeneities between centers. However, limitations of this study should be mentioned. First, information on the primary cooking and heating source were acquired using self-reported questionnaires which is not an objective measurement and may only reflect the participants’ adoption during baseline survey. Second, the inclusion of people receiving antihypertensive medication in the analysis may potentially introduce a risk of selection bias, because their blood pressures were under control. We conducted sensitivity analyses according to participants’ statuses on receiving antihypertensive medications and the findings were robust. Third, we did not include season in the final model, however, as season is correlated with the heating provision period, including season in the model may seriously underestimate the risk estimate of interest related to household fuel use for heating. Concerns may also arise from a relatively small group using electricity or liquid (kerosene) particularly when further subgroup analyses by ambient PM2.5 and location were performed, which might yield the effect to be measured in an unstable way. This limitation along with the nature of cross-sectional study of the baseline survey made the cause-effect difficult to determine, and thus the associations would have to be confirmed by prospective cohort studies involving repeated measurement of blood pressure and hypertension during the follow-up. Given the actual pattern of ambient PM2.5 exposure level in mainland China, we used the median ambient PM2.5 concentration of 45.8 ug/m^3^ rather than WHO’s annual standard as the cut-off to group the high and low exposure groups to keep a reasonable sample size and power in the subgroup analysis. Lastly, while the baseline data were collected during 2005–2009, the results are still relevant to some remote rural areas in China and the developing countries in which the unclean fuels are still being used.

### Conclusion

In conclusion, this study uniquely provides evidence that utilizing cleaner types of household fuel is associated with lower hypertension prevalence and a lower level of blood pressure in Chinese adults, with a more pronounced association among people with high ambient PM2.5 exposure. Electricity, ranked as the cleanest fuel type, was associated with the lowest hypertension prevalence and the lowest level of blood pressure compared with the uses of other household fuel types. Gas, ranked as a highly clean level on the energy ladder, was also associated with lower hypertension prevalence and lower blood pressure values, but the effect was weaker than that of the household electricity users for cooking and heating. Our study urges the promotion of cleaner household fuel usage to reduce the burden of hypertension and therefore the subsequent complications induced by hypertension.

## Data Availability

The datasets analysed during the current study are not publicly available due to an ongoing project, but are available from the corresponding author on reasonable request.

## Abbreviations

AOR, Adjusted odds ratio; BMI, Body mass index; CI, Confidence interval; DBP, Diastolic blood pressure; DHS, Demographic and Health Surveys; IQR, interquartile range; OR, Odds ratio; PURE, the Prospective Urban and Rural Health Evaluation Study; PM 2.5: Particulate Matter 2.5; SBP, Systolic blood pressure; SD, standard deviation.
